# Cases Illustrating the Practical Use of the Opthalmoscope, as a Means of Diagnosis in Certain Diseases of the Eye

**Published:** 1859-04

**Authors:** E. L. Holmes

**Affiliations:** Chicago, Ill.


### ARTICLE II.

### CASES ILLUSTRATING THE PRACTICAL USE OF THE OPHTHALMOSCOPE, AS A MEANS OF DIAGNOSIS, IN CERTAIN DISEASES OF THE EYE.

BY E. L. HOLMES, M.D., OF CHICAGO, ILL.

[Continued from page 141.]

*Case III. Incipient Cataract.*—A gentleman, who had been struck upon the right eye with a riding whip, came to me some time after the injury, for examination, stating that, since the blow, the distinctness of vision in this eye had been much diminished, and that any continued use of the eyes was attended with great discomfort. The general aspect of the eye was normal, with the exception of a slight dilatation of the pupil, which had existed from the first. No belladonna had been applied to the eye, as I was informed by the patient.

An examination with the ophthalmoscope, disclosed upon the posterior surface of the lens a dark line, extending from near the centre downwards, and inwards to the periphery. This line resembles one of the radii, which are usually found in soft cataracts, as delineated in most works upon diseases of the eye. Usually there are several radii observed in the affected lens, and it is quite rare that even in incipient cataract, only a single line of opacity is found. The transparent media of the eye in other respects are normal.

It is not my object to discuss the effects of the blow, but simply to describe the condition of the eye as I found it. There are many reasons for believing that the cataract in this case had existed for a longer period than the patient imagined, and that the blow called his attention to the fact that vision of this eye was imperfect. Cases are not altogether uncommon where patients have suddenly discovered that vision of one eye was wholly lost, upon making an attempt to look with one eye closed.

*Case IV. Photopsia.*—Patient is an Irish woman, aged forty years, of full habit, and addicted to the use of whisky. For a long time she has been troubled with occasional ringing in the right ear, and with symptoms of congestion of the nervous apparatus of both eyes, being annoyed by brilliant flashes of different colored lights; appearing in the field of vision, sometimes in the form of spheres, and at others without any defined figure. Patient often imagines she sees dark irregular flocula, resembling tufts of black cotton floating before the eyes. These appearances are all entirely *subjective*, since they seem to float before the patient's eyes in various directions independent of the motions of the globes. Floating bodies in a liquified vitreous humor, usually take a sudden motion upwards; on turning the globes quickly upwards, again to sink to their former position. Bodies fixed in a position directly behind the pupil always appear, as far as I know, in the same relative point in the field of vision.

There is scarcely any modification in the condition of the iris. Vision is still perfect, except during the paroxysms of photopsia, when patient finds difficulty in obtaining a distinct image of distant objects. The conjunctiva, as also all the other membranes visible to the eye, are healthy and free from congestion.

The retina of each eye, especially around the entrance of the optic nerve, presents, when viewed through the opthalmoscope, an appearance of congestion, this membrane being of a darker red, and the papilla of the nerve being less well defined than natural. In all probability, these appearances are sufficient to account for the subjective symptoms which annoy the patient, since similar changes in the color of the retina are often accompanied with photopsia.

In the right eye, moreover, which gives the patient the most trouble, there is a commencement of the process described in case number two, there being a slight deposition of pigment matter in the external and anterior portion of the concavity of the eye.

I am disposed to believe that this patient might be relieved of her symptoms in a measure, by suitable treatment. She is, however, in a miserable condition, exposing herself by every kind of irregularity to an increase of the disease, and neglecting to follow any course of treatment.

*Case V. Encephaloid Disease of the Retina.*—Some time since, I examined a boy, aged six years, to all appearance enjoying perfect health, who, for two or three months, had complained of increasing disturbance of vision in the left eye, the other being unaffected. Except a considerable dilatation of the pupil, the eye appeared in all respects normal. The patient could not, however, distinguish objects in the least with this eye, and was scarcely conscious of even bright light falling directly into the pupil. At this stage of the disease, I think, no one could have made a satisfactory diagnosis without the assistance of the ophthalmoscope. With it the posterior portion of the ocular cavity could be seen, beginning to present the peculiar appearance of encaphaloid disease of the eye (*retina ?*) which needs only to be seen once to be remembered. The diseased portion embraced about a quarter of the retina around the optic nerve, which it also involved. The peculiar, brilliant light copper-colored tint was already somewhat developed, and vessels were observed running in beautiful branches over the carious substance, which was still very thin, having as yet scarcely invaded the cavity of the eye.

Several readers of the *Journal* will recollect an example of this fearful disease, which I examined with them a few months since, in the case of an infant, in which the disease was so far developed in both eyes, that there could be no doubt, as regards diagnosis, even with the unassisted eye. In the right eye, the disease had wholly filled the cavity of the eye behind the iris.

Unfortunately, the only benefit gained by the use of the ophthalmoscope in the case just reported, was simply the diagnosis, which, with this instrument, could have been made even earlier; and even the sad revelation itself might, under the circumstances, prove of advantage to the sufferer, since he would be saved the trouble and pain of undergoing the infliction of the usual treatment for *amaurosis*, by purging, salivation, blistering, and cupping, which could only torment the patient without affecting the disease.

To me these three cases are of great interest, in illustrating the practical use of the ophthalmoscope in detecting the commencement of processes, which would remain wholly concealed from view without it.

[Concluded in next number.]

